# Supplementary figures and images for: Serial histological changes in the cartilaginous eustachian tube in the rat following balloon dilation
Source: PLoS One. 2022 May 25;17(5):e0268763. doi: 10.1371/journal.pone.0268763 (PMC9132338; doi:10.1371/journal.pone.0268763)

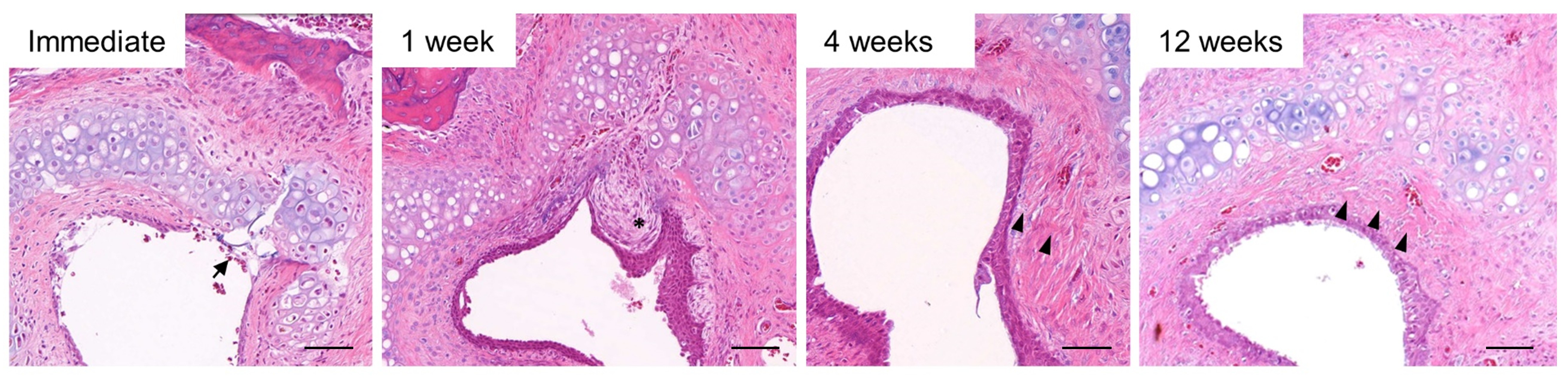

Supplement: S1 Fig — Mucosal breaks are observed immediately after ETBD (arrow). Mucosal wound is healed by 1 week and fibroblasts (asterisk) are seen. At 4 and 12 weeks, the submucosa is replaced with collagen tissue (arrowheads). Scale bars represent 100μm. (TIF) [file pone.0268763.s001.tif]
