# Supplementary material for: Serial histological changes in the cartilaginous eustachian tube in the rat following balloon dilation
Source: PLoS One. 2022 May 25;17(5):e0268763. doi: 10.1371/journal.pone.0268763 (PMC9132338; doi:10.1371/journal.pone.0268763)
Supplement: Attachment — Submitted filename: Response to Reviewers.docx [file pone.0268763.s003.docx]

We appreciate the reviewers’ valuable comments that potentially improve our manuscript. We made our best effort to reflect the suggestions in the revised manuscript. Below in blue are our line-by-line responses to the reviewers’ comments.

Reviewer #1: The manuscript entitled: “Serial histological changes in the cartilaginous Eustachian tube in the rat following balloon dilation” is interesting and purposeful in the aspect of understanding processes that occur in the tissues after Eustachian tube ballon dilation. The Authors have assessed the epithelium, submucosa and the lumen of the ET using a basic histological technique enriched with morphometric analyses.

The Authors have put a great effort to improve their manuscript after first revision. My comments and suggestions were taken into consideration, however there are still some minor issues that should be revised:

1. In References section, literature positions 21 and 24 are the same. After deleting one of them the numbers in the text should be corrected.

→ Thank you for pointing this out. Reference 24 was removed and changed to 21.

2. In References some names of journals are still not abbreviated:

Reference 25: J Vasc Interv Radiol

Reference 29: J Pharm Bioallied Sci

Reference 34: Int J Pediatr Otorhinolaryngol

Reference 38: Am J Otolaryngol

→ Thank you for pointing this out. The abbreviations were corrected as suggested.

3. Figures and Figure legends

In Fig 3 and 4 the white arrowheads are barely visible

→ For Fig 4, the white arrowheads were changed to “white arrowheads with black borders” for better visualization. For Fig 3, there was a mistake with the legends so the sentence reading “White arrowheads denote blood vessels” was removed from the manuscript.

After * in fig 5 and 6 there should be: *statistically significant differences for p<0.05, Mann-Whitney U test.

→ Thank you for the suggestion. The suggested corrections were made to the manuscript.

In S1 Fig. there should be a scale bar or magnification indicated in the legend.

→ Scale bars were added to the figure and indicated in the legend

In my opinion, the manuscript is suitable for publication in PLOS ONE after minor revision.

→ Thank you for your positive feedback.

Reviewer #2: The work has been corrected in accordance with the comments of the reviewer, but I do have some remark, however. I see ambiguity in the description of Figure 4 (lines 457-460) and the description of the Results (lines 190-192) - what exactly did the authors want to write - epithelial hyperplasia or squamous metaplasia?

→ There was confusion with the choice of terminology in the original manuscript. Thank you for pointing this out. In the new manuscript, the finding was uniformly described as epithelial hyperplasia.
